# Bilateral Perivascular Chorioretinal Atrophy Resembling Pigmented Paravenous Chorioretinal Atrophy Post COVID-19 Infection: A Case Report and Comprehensive Immune Profiling

**DOI:** 10.3390/vaccines12080878

**Published:** 2024-08-02

**Authors:** Tomohito Sato, Yuki Takenaka, Masaru Takeuchi

**Affiliations:** Department of Ophthalmology, National Defense Medical College, 3-2 Namiki, Tokorozawa 359-8513, Saitama, Japan; dr21043@ndmc.ac.jp (T.S.); mii.52yy@gmail.com (Y.T.)

**Keywords:** COVID-19, cytokine, hierarchical cluster analysis, optical coherence tomography, pigmented paravenous chorioretinal atrophy, mass cytometry, multiplex bead analysis

## Abstract

The pandemic of COVID-19 caused by the SARS-CoV-2 virus is ongoing and a serious menace to global public health. An ocular manifestation is an initial sign of the infection. To date, a comprehensive immune profile of patients with mild COVID-19 has not been well developed. Here, we report a 53-year-old female who noticed a sudden decrease in visual acuity (VA) in both eyes on the fourth day after COVID-19 infection. At presentation (acute phase), the best-corrected VA (BCVA) on the decimal chart was 0.5 in both the right and left eyes. Color fundus photography showed perivascular chorioretinal atrophy with peripheral pigment loss, similar to the fundus appearance of pigmented paravenous chorioretinal atrophy (PPCRA) in the inferior arcade vessels of both eyes. Optical coherence tomography indicated thinning and blurred boundaries of the outer retina in the lesion sites, implying anatomical destruction. She was followed up without any systemic medications. After approximately 15 weeks (remission phase), the BCVA recovered to 0.6 in the right eye and 0.8 in the left. Systemic immune profiles were analyzed using mass cytometry. In the acute phase, monocytes and basophils were dominantly elevated, which suggested the activation of innate immune responses to SARS-CoV-2 and allergic inflammation. In the remission phase, Th2-like cells, plasmablasts, and neutrophils increased predominantly, implying the maturation of adaptive immunity and the preparedness of innate immunity to combat the infection. Our findings indicate that perivascular chorioretinal atrophy resembling PPCRA is a clinical feature of the ocular phenotype of COVID-19, caused by systemic immune responses.

## 1. Introduction

The pandemic of coronavirus disease 2019 (COVID-19) caused by severe acute respiratory syndrome coronavirus 2 (SARS-CoV-2) is ongoing [1] and a serious menace to global public health. COVID-19 leads to respiratory dysfunction as well as a systemic thromboembolic state that induces serious cardiovascular, cerebrovascular, and peripheral vascular manifestations [1]. In epidemiological surveys of COVID-19 outbreaks from 2019 to 2020 [2,3], although the infection rate of COVID-19 varies greatly depending on the country and region, approximately 30% to 50% of patients infected with COVID-19 are asymptomatic and 20% develop severe symptoms. Five percent of the severe patients also require mechanical ventilation, and half of those on ventilation die. Similarly to other human respiratory coronaviruses, SARS-CoV-2 appears to exhibit neuroinvasive and neurotropic activity [4,5]. In a retrospective case series study, Mao et al. [5] proposed that COVID-19-related neurological symptoms could be categorized into three groups: central nervous system (CNS) manifestations, peripheral nervous system (PNS) symptoms, and musculoskeletal disorders. Hyposmia has been reported as a potential PNS symptom associated with COVID-19 infection [5], and Bonzano C et al. [6] suggested that smell alteration (hyposmia, anosmia) may be one of the earliest manifestations of COVID-19, often occurring with or without the loss of taste (dysgeusia).

Ocular manifestations during COVID-19 infection, including conjunctivitis, retinal vascular occlusions, and optic neuritis, have been reported [1,7]. Retinal manifestations may occur from within a week after the onset of COVID-19 symptoms to after more than 6 weeks [1]. The occurrence of vision-threatening ocular symptoms caused by SARS-CoV-2 is rare but a serious concern, although the immunopathology of the ocular manifestations remains unclear.

The eye is a unique organ that allows light to pass through. Therefore, the anatomy of the retina located at the fundus can be observed in detail and over time. Recent reports have documented cases of COVID-19–associated retinopathy [1]. However, the roles of immune cells and their comprehensive involvement in COVID-19 pathogenesis have not been well addressed [8]. Here, we report a case of bilateral perivascular chorioretinal atrophy similar to the fundus appearance of pigmented paravenous chorioretinal atrophy (PPCRA) in a post-COVID-19 patient, and we further analyze the immune profiles of the patient in both the acute and remission phases using multiplex bead analysis and mass cytometry by time-of-flight (CyTOF) technology.

## 2. Case Presentation

A 53-year-old Japanese woman was referred to our hospital (2X August 2022) for an investigation of vision loss after the onset of COVID-19. Fifteen days before her transfer, the patient presented to a local doctor with a sore throat and was diagnosed with COVID-19 by real-time reverse transcription polymerase chain reaction (RT-PCR) testing for SARS-CoV-2 ribonucleic acid (RNA) using a nasopharyngeal swab. On the fourth day after symptom onset, she experienced a sudden decrease in visual acuity (VA) in both eyes, coinciding with the resolution of fever. She was then referred to our hospital. Her medical history revealed that she had received three doses of mRNA COVID-19 vaccine (BNT162b2; Pfizer-BioNTech, Kronach, Germany). The last dose was administered approximately seven months before the current illness. At the time of the patient’s presentation, the predominant SARS-CoV-2 variants in Japan may the BA.5 lineages and their sublineages of the Omicron variant, reported by the National Institute of Infectious Diseases of Japan [9,10].

**Figure 1:**
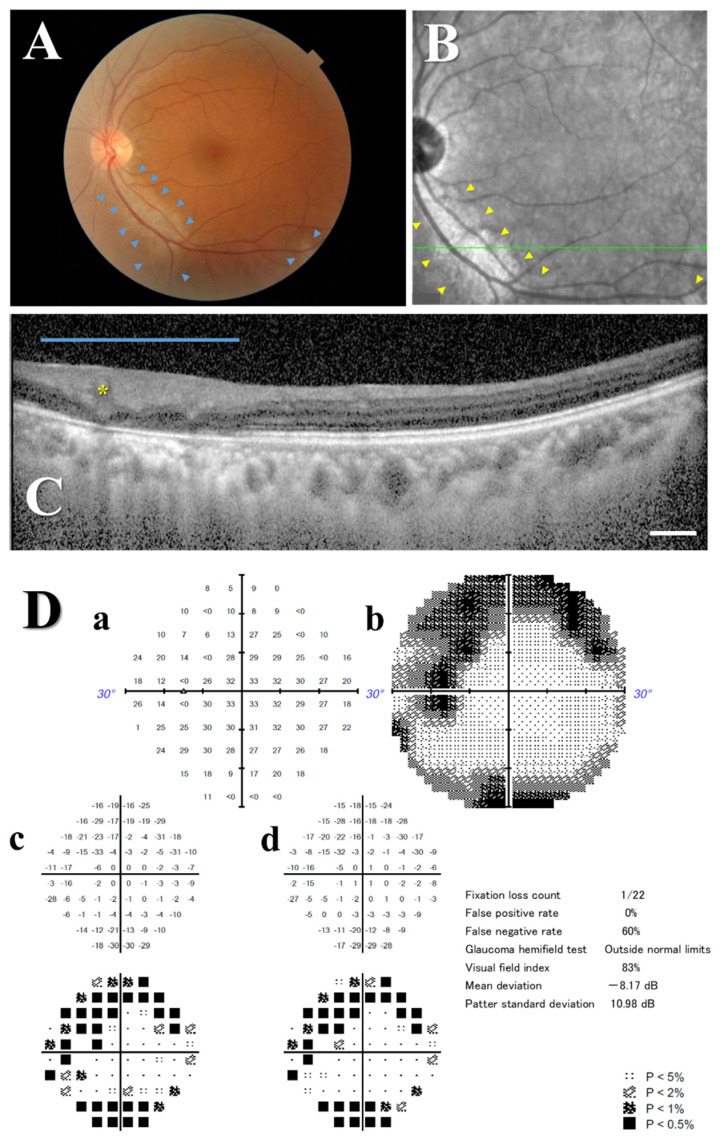
Fundus findings and visual field results of the left eye in the acute phase. (**A**) Color fundus photograph shows absence of retinal pigment along the inferior arcade vessels extending from the disk in the posterior retina (areas enclosed by blue arrowheads), suggesting perivascular chorioretinal atrophy associated with COVID-19. (**B**) Red-free fundus photograph and (**C**) cross-sectional EDI-OCT image of the retina along the green line. Retinal lesion is enclosed by yellow arrowheads. The blue bar indicates the extent of the lesions, and the yellow asterisk highlights a retinal blood vessel. The outer retinal layers are thinned, and the retinal layers are indistinguishable. The ellipsoid zone (photoreceptor layer) in the outer retinal layer is absent. Scale bar (white horizontal bar): 200 μm. (**D**) A Humphrey 30-2 SITA-Standard visual field test demonstrates scotomas in the superior temporal quadrant corresponding to the lesions with a mean deviation of −8.17 dB. (**a**) Numerical sensitivity plot, (**b**) grayscale map, (**c**) total deviation map, and (**d**) pattern deviation map are shown. COVID-19: coronavirus disease 2019; EDI-OCT: enhanced depth imaging optical coherence tomography; SITA: Swedish interactive thresholding algorithm.

On presentation (the acute phase), her best-corrected visual acuity (BCVA) measured using a decimal chart was 0.5 (logMAR 0.30) in both the right and left eyes. Slit-lamp examination revealed no inflammatory cells in the anterior chamber or anterior vitreous cavity in either eye. The flare values in the anterior chamber averaged over ten measurements were 2.4 photons/ms in the right eye and 2.1 photons/ms in the left eye. Color fundus photography and fundus autofluorescence imagery demonstrated atrophic changes in the retinal pigment epithelium (RPE) along the inferior arcade vessels extending from the disk to the mid-peripheral retina in both the left eye (Figure 1A and Supplementary Figure S3A) and the right eye (Supplementary Figures S1A and S2A). Enhanced depth imaging optical coherence tomography (EDI-OCT) revealed the thinning of the outer retina and blurring between the outer retinal layers in the lesion sites, suggesting disruption of the retinal architecture, in the left eye (Figure 1B,C) and the right eye (Supplementary Figure S1B,C). A Humphrey visual field test demonstrated scotomas (depressions) corresponding to the retinal lesions in the left eye (Figure 1D) and the right eye (Supplementary Figure S1D). Fluorescein angiography (FA) revealed diffuse punctate hyperfluorescence at the areas of RPE loss, indicating RPE damage or dysfunction, without significant retinal vascular leakage or perfusion defects in the right eye (Supplementary Figure S2E) and the left eye (Supplementary Figure S3E). To rule out other infectious causes of retinitis or endophthalmitis, an aqueous humor (AH) sample collected from the left eye in the acute phase was analyzed using a comprehensive polymerase chain reaction (PCR) panel [11]. The PCR panel detected no evidence of the following pathogens: human herpesviruses 1 to 8, bacteria (16S ribosomal RNA [rRNA]), fungi (28S rRNA), syphilis, tuberculosis, toxoplasmosis, and toxocariasis. The patient was subsequently managed without systemic treatment.

At approximately 15 weeks after the initial presentation (the remission phase), the BCVA had improved to 0.6 (logMAR 0.22) in the right eye and 0.8 (logMAR 0.10) in the left eye. Visual field defects were partially resolved in the right eye (Supplementary Figure S4D) and the left eye (Supplementary Figure S5D). The anatomical structure of the outer retinal layers had recovered and become distinguishable by EDI-OCT (right eye: Supplementary Figure S4C; left eye: Supplementary Figure S5C).

## 3. Comprehensive Immune Profiling

### 3.1. Aqueous Humor and Serum Cytokine Profiles in Acute Phase

To investigate the patient’s immune status, we collected blood samples and residual aqueous humor (AH) in the acute phase (P-Onset: 3X August 2022) and the remission phase (P-Remission: 2X December 2022). Three healthy volunteers (No. 1: 45-year-old female, No. 2: 51-year-old male, No. 3: 56-year-old female) were recruited from our hospital staff as controls. The levels of 27 cytokines in AH and serum were measured using a multiplex bead analysis system (Bio-Plex Human Cytokine 27-plex panel; Bio-Rad, Hercules, CA, USA) [12] (Table 1). CyTOF (Helios™, Fluidigm, South San Francisco, CA, USA) with a Maxpar Direct Immune Profiling Assay^®^ (Fluidigm) [13] were used to analyze the phenotypes and proportions of immune cells in peripheral blood leukocytes (Table 2). This method allows for the classification of leukocytes into 37 distinct immune cell types based on differentiation, function, and maturation stage [14].

In P-Onset, the AH levels of interleukin (IL)-1 receptor antagonist (ra), IL-6, IL-12, macrophage inflammatory protein (MIP)-1β, and vascular endothelial growth factor (VEGF)-A were 13.8 pg/mL, 1.58 pg/mL, 0 pg/mL, 6.54 pg/mL, and 0 pg/mL, respectively. Compared to the published reference ranges for AH cytokines [12], IL-1rα was relatively high, while IL-6, IL-12, MIP-1β, and VEGF-A were relatively low. These findings suggest an immunosuppressive response in the context of perivascular chorioretinal atrophy. For serum cytokines, IL-1ra, IL-8, IL-9, eotaxin, granulocyte colony-stimulating factor, interferon-γ, interferon gamma-induced protein 10, monocyte chemotactic protein-1, and regulated on activation, the normal T cells expressed and secreted were 62.0 pg/mL, 2.87 pg/mL, 42.2 pg/mL, 75.7 pg/mL, 127.5 pg/mL, 2.29 pg/mL, 303.1 pg/mL, 7.43 pg/mL, and 564.0 pg/mL, respectively. Compared to the control group and published reference ranges [14], the levels of these serum cytokines were relatively elevated, suggesting a heightened systemic immune response to SARS-CoV-2 [8], in contrast to the immunosuppressive profile of AH cytokines.

### 3.2. Hierarchical Cluster Analysis

**Figure 2:**
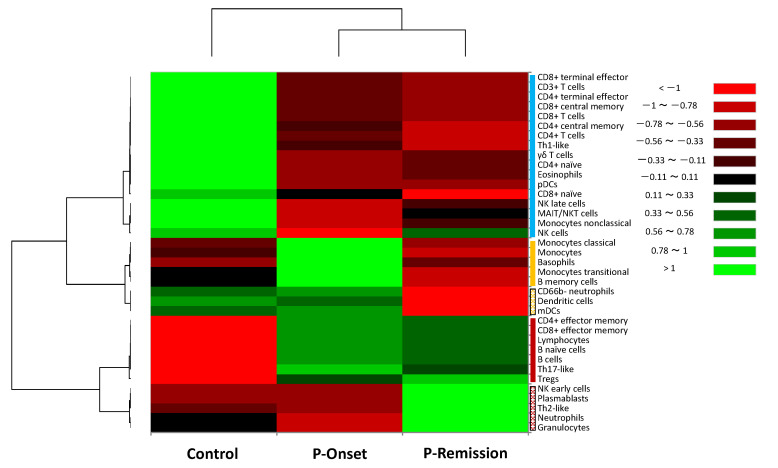
Hierarchical cluster analysis of immune cell populations and phenotypes in peripheral blood. The heatmap depicts the classification of 37 immune cell populations and phenotypes among leukocytes in the peripheral blood of the patient in the acute phase (P-Onset) and remission (P-Remission), and in the control group. The analysis identified three principal clusters based on property similarity: Cluster A (blue bar), Cluster B (yellow bar; divided into Subclusters B1 [filled bar] and B2 [patterned bar]), and Cluster C (red bar; divided into Subclusters C1 [filled bar] and C2 [patterned bar]). The color scale represents expression levels, with red indicating low values and black to green indicating progressively higher values. The vertical axis lists the immune cell populations and phenotypes, while the horizontal axis shows the patient in the acute and remission phases, and the control group. P-Onset: the patient in the acute phase; P-Remission: the patient in the remission phase.

Hierarchical cluster analysis was performed to classify peripheral blood leukocytes into distinct groups based on property similarity, termed clusters [15]. The leukocytes in P-Onset, P-Remission, and controls were broadly categorized into three principal clusters (Figure 2): (1) Cluster A (blue bar), (2) Cluster B (yellow bar), and (3) Cluster C (red bar).

Cluster A was composed of terminal effector cluster of differentiation (CD) 8^+^ T cells, CD3^+^ T cells, terminal effector CD4^+^ T cells, central memory CD8^+^ T cells, CD8^+^ T cells, central memory CD4^+^ T cells, CD4^+^ T cells, T helper (Th)1-like, γδ T cells, naïve CD4^+^ T cells, eosinophils, plasmacytoid dendritic cells (pDCs), naïve CD8^+^ T cells, natural killer (NK) cells late, mucosal-associated invariant T/natural killer T cells, non-classical monocytes, and NK cells. The expression level of this cluster was high in the control group compared to P-Onset and P-Remission. Cluster B consisted of a subcluster (B-1, filled yellow bar) comprising classical monocytes, monocytes, basophils, transitional monocytes, and memory B cells, and another subcluster (B-2, patterned yellow bar) consisting of CD66b^−^ neutrophils, dendritic cells (DCs), and myeloid DCs. Cluster C also contained two subclusters. Subcluster C-1 (filled red bar) was composed of effector memory CD4^+^ T cells, effector memory CD8^+^ T cells, lymphocytes, naïve B cells, B cells, Th17-like, and regulatory T cells. Subcluster C-2 (patterned red bar) consisted of NK cells early, plasmablasts, Th2-like, neutrophils, and granulocytes.

The expression level of subcluster B-1 was elevated in P-Onset compared to P-Remission and the control group. This suggests a characteristic feature of systemic immune responses to COVID-19 and allergic inflammation. The expression level of subcluster C-2 was elevated in P-Remission compared to P-Onset and the control group, implying maturation of the systemic immune response against SARS-CoV-2. Interestingly, subcluster C-1 remained persistently elevated during both P-Onset and P-Remission. This may indicate a sustained activation of adaptive immune responses, particularly those involving Th17 cells.

### 3.3. Other Data

Hematological data for P-Onset and P-Remission are detailed in Supplementary Tables S1 and S2, respectively. Serum cytokine levels for each control subject are provided in Supplementary Table S3. Supplementary Table S4 presents the profile of leukocyte phenotypes and the proportions of the phenotypes in the peripheral blood of each control subject.

## 4. Discussion

The ongoing COVID-19 global pandemic has resulted in millions of deaths and a wide range of severe clinical manifestations including post-recovery complications [1]. The typical clinical course of COVID-19 often begins with a fever, dry cough, and malaise, progressing to a mild-to-moderate lower respiratory illness that resolves without specific treatment in most cases [16]. However, a significant proportion of patients can develop neurological and vascular complications [17,18]. These patients may present major systemic immune dysfunction leading to multiorgan failure [19]. Notably, ocular manifestations can be the initial sign of COVID-19 [20], with acute conjunctivitis being the most common presentation [21].

Several pathways by which SARS-CoV-2 enters the central nervous system (CNS) have been proposed. One involves hematogenous spread. The virus enters the CNS via the bloodstream and may affect the capillary endothelium due to slower blood flow in these regions [22,23]. Notably, the retina shares embryonic origins with the optic nerve, which is considered the second cranial nerve and thus part of the CNS [24]. Recent case reports describe SARS-CoV-2-mediated injury to the neurosensory retina and optic nerve [1]. Furthermore, detection of SARS-CoV-2 RNA in the retina obtained at autopsy of COVID-19 patients provides additional evidence for a potential retinal involvement [25]. Based on these findings, a hypothesis has emerged suggesting that SARS-CoV-2 may cause various retinal complications, which may lead to loss of vision as well [1]. However, reports of retinal complications directly attributable to SARS-CoV-2 infection remain scarce [1,26].

PPCRA is an uncommon form of chorioretinal atrophy characterized by perivenous aggregations of pigment clumps associated with peripapillary and radial zones of RPE atrophy that are distributed along the retinal veins [27,28]. It typically presents bilaterally and symmetrically [27]. When present, the most common symptoms are a mild visual loss, reduction in the peripheral visual field, and nyctalopia [27,28,29,30]. While patients affected by PPCRA are typically asymptomatic, the diagnosis is established on the basis of its distinctive fundus appearance [27,28,29]. The natural history of PPCRA is either non-progressive or slowly progressive [27]. Pigmentary alterations can range from fine to coarse clumping, resembling bone corpuscles [28]. Although the etiology of the disease is unknown, various inflammatory and infectious etiologies including uveitis, Vogt–Koyanagi–Harada disease, Behcet’s disease, congenital syphilis, tuberculosis, neurofibromatosis type 1, and viral infections (measles, rubella) have been proposed [27,29]. Differential diagnoses include other chorioretinal degenerative and inflammatory diseases causing chorioretinal atrophy such as pericentral, sectorial, and typical retinitis pigmentosa, helicoid peripapillary chorioretinal atrophy, serpiginous choroidopathy, autoimmune retinopathy, hydroxychloroquine retinopathy, sarcoidosis, gyrate atrophy, choroideremia, tuberculous choroiditis, toxoplasmosis, cone dystrophy, syphilis, and angioid streaks [27,29]. To date, no cases linking PPCRA-like fundus findings to vaccine injection or COVID-19 infection have been reported. In this case, the fundus findings were similar to the appearance of PPCRA, except for the absence of retinal pigment. The depigmented appearance suggests the possibility of early-stage PPCRA. This hypothesis will require a longer follow-up to confirm.

Additionally, an experimental murine coronavirus retinopathy model demonstrates a biphasic development of virus-induced retinopathy [31]: an early phase and a late phase. The early phase is characterized by retinal inflammation, immune cell infiltration, and release of inflammatory mediators. Viral clearance typically begins after the first week of infection. The late phase involves the production of autoantibodies against the retina and RPE cells, leading to photoreceptor and neuroretinal damage. Based on these mechanisms, the perivascular chorioretinal and RPE atrophies observed in this case may be caused by SARS-CoV-2 via the pathological mechanisms mentioned above. The comprehensive roles of leukocytes in the systemic immune response to COVID-19 remain unclear [8]. This case report aimed to examine the immune profile of peripheral blood leukocytes by mass cytometry in a patient with mild COVID-19 presenting with retinal vasculitis.

For CyTOF analysis, we employed hierarchical cluster analysis to comprehensively evaluate the property similarities among leukocyte populations in P-Onset, P-Remission, and healthy controls. Our study confirmed elevated serum levels of inflammatory cytokines including IL-8 (Table 1), and a dominance of innate immune responses including elevated monocytes (Figure 2) in the acute phase. These findings are consistent with the results of a cohort study of COVID-19 patients hospitalized at the Mount Sinai Health System in New York, which showed elevated serum levels of IL-6, IL-8, and TNF-α on admission, and patients with higher cytokine levels exhibited greater disease severity and poorer survival rates [32]. In addition, other studies have reported marked decreases in circulating pDCs, NK cells, and CD8^+^ T cells in the acute phase of COVID-19 [33,34]. Overall, our findings of the immune dynamics in the acute phase of a patient with mild COVID-19 are generally consistent with previous reports.

In this study, a comprehensive PCR panel [11] was employed to rule out the co-occurrence of potential bacterial or viral infections. However, the PCR panel is limited in its ability to assess the presence of unknown microorganisms, because it amplifies and extracts only specific DNA sequences. Metagenomics is a new high-yield DNA sequencing method to provide taxonomic and functional profiles of microbial communities without the need to culture microbes in the laboratory [35]. Borroni D, et al. [36] employed shotgun metagenomics analysis to evaluate the microbiota of culture-negative corneal impression membranes in microbial keratitis samples. They successfully identified putative pathogens in all 18 samples, even those that eluded detection by conventional culture methods, and further quantified their relative abundances. Therefore, it seems that metagenomics analysis holds immense promise as a next-generation method for broad-spectrum infection assessments, particularly in the diagnosis of challenging cases.

The patient’s visual acuity remained generally good throughout the acute phase and was preserved during remission. Notably, no clinical manifestations were observed in other organs. Consequently, the patient’s clinical course was monitored without the need for systemic medications. A key strength of our CyTOF analysis lies in the ability to reveal the evolution of the immune profile from the acute phase to remission in a treatment-naive patient with mild COVID-19.

This study had some limitations. First, while prior vaccination with three doses of BNT162b2 vaccine could theoretically induce an exaggerated immune response to COVID-19, existing evidence suggests that this is unlikely. A test-negative design study conducted by Israel et al. [37] showed no significant difference in the odds ratio of a positive RT-PCR test for COVID-19 more than 90 days after the second dose compared to less than 90 days, in adults who received two doses and had no prior infection with COVID-19. These findings suggest that the influence of previous vaccinations on the observed immune profile in our patient may have been minimal. Second, the immune response in COVID-19 patients is known to vary depending on disease severity [8,38]. Third, the validity of the serum cytokine data obtained from controls remains uncertain due to the absence of well-defined reference intervals for each cytokine. To establish reliable reference intervals, it would be appropriate to analyze serum samples from at least 20 healthy blood donors in a medical facility. Due to the inherent limitations of a single case report with a control group drawn from a restricted population, the generalizability of our findings to the broader population of mild COVID-19 cases remains constrained.

## 5. Conclusions

Ocular manifestations can be an initial sign of COVID-19 [20], and bilateral perivascular chorioretinal atrophy is documented to be a rare ocular complication of the disease. The immune profiles during the acute and remission phases of patients with mild COVID-19 remain poorly understood. In this study, we employed multivariate analysis using CyTOF to classify leukocytes based on property similarity, revealing distinct immune dynamics depending on the disease stage. Further studies and case reviews are necessary to comprehensively elucidate the immune mechanisms underlying COVID-19-related complications. Notably, our case provides valuable insights into the clinical ocular findings and systemic immunological shift observed in a patient with mild COVID-19, potentially contributing to deeper understanding of the pathology of this disease.

## Figures and Tables

**Table 1:** Cytokine levels in serum and aqueous humor from the left eye in the acute phase.

	Patient		Controls			Detection Range	
*n*			3				
Cytokine	AH	Serum	Serum			Lower	Upper
			Median	First Quartile	Third Quartile		
PDGF-BB	0	443.4	572.1	327.9	723.2	10.5	42,619
IL-1β	0	0	0	0	0.16	0.34	5037
IL-1rα	13.8	62.0	24.2	12.1	26.0	8.56	37,277
IL-2	0	0	0	0	0	1.63	7740
IL-4	0	1.98	1.92	1.10	2.56	0.17	3540
IL-5	0	0	0	0	0	6.02	85,349
IL-6	1.58	0	0	0	0	0.98	3825
IL-7	7.74	0	0	0	0	1.84	11,702
IL-8	0.68	2.87	1.30	0.65	1.74	0.51	10,416
IL-9	0	42.2	29.9	14.9	37.5	2.11	10,043
IL-10	0	0	0	0	0	2.77	12,756
IL-12	0	0	0	0	0	1.58	21,263
IL-13	0	0	0	0	0	0.75	3908
IL-15	0	0	0	0	0	559.4	76,423
IL-17A	0	0	0	0	1.53	2.65	35,294
Eotaxin	1.37	75.7	7.60	6.40	71.1	0.20	787
bFGF	0	0	0	0	2.34	5.45	5446
G-CSF	0	127.5	43.8	21.9	77.4	57.3	6793
GM-CSF	0	0	0	0	0	0.33	1534
IFN-γ	2.82	2.29	0	0	0	1.20	22,826
IP-10	205.2	303.1	70.5	59.4	106.3	1.53	23,607
MCP-1	158.7	7.43	3.35	1.68	5.17	0.34	5762
MIP-1α	0.32	1.30	0.40	0.20	0.42	0.05	49.0
MIP-1β	6.54	23.4	20.1	11.8	21.2	0.46	2126
RANTES	2.66	564.0	150.0	89.3	269.2	1.00	3499
TNFα	0	0	0	0	2.04	4.07	18,347
VEGF-A	0	0	0	0	0	19.5	61,637

Cytokine concentrations are expressed as pg/mL. Levels below the detectable limit were assigned a value of zero [14]. AH: aqueous humor; bFGF: basic fibroblast growth factor; G-CSF: granulocyte colony-stimulating factor; GM-CSF: granulocyte macrophage colony-stimulating factor; IFN-γ: interferon-gamma; IL: interleukin; IL-1ra: IL-1 receptor antagonist; IP-10: interferon gamma-induced protein 10; MCP-1: monocyte chemotactic protein-1; MIP: macrophage inflammatory protein; PDGF: platelet-derived growth factor; RANTES: regulated on activation, normal T-cell expressed and secreted; TNFα: tumor necrosis factor alpha; VEGF: vascular endothelial growth factor.

**Table 2:** Immune cell populations, phenotypes, and proportions of immune cells among leukocytes in the peripheral blood of the patient in the acute and remission phases, and in controls.

Populations *n* Disease Stage					Model Phenotypes	Patient		Controls		
						Acute	Remission	3		
								Median	First Quartile	Third Quartile
Intact live cells (%)										
	Lymphocytes				CD3 T cells + B cells + NK cells + plasmablasts	64.7	64.3	59.8	53.3	68.3
		CD3^+^ T cells			CD8 T cells + CD4 T cells + γδ T cells + MAIT/NKT cells	30.8	29.4	44.8	36.7	49.1
			CD8^+^ T cells		CD3+ CD66b- CD19- CD8+ CD4- CD14- CD161- TCRγδ- CD123- CD11c-	7.50	6.84	10.74	9.44	17.8
				*Naïve*	CD8 T cells + CD45RA+ CCR7+ CD27+	2.29	2.26	2.32	1.97	3.22
				*Central memory*	CD8 T cells + CD45RA- CCR7+ CD27+	0.05	0.04	0.12	0.11	0.18
				*Effector memory*	CD8 T cells + CCR7- CD27+	3.99	3.71	2.04	1.94	2.78
				*Terminal effector*	CD8 T cells + CCR7- CD27-	1.18	0.83	4.59	4.53	11.7
			CD4^+^ T cells		CD66b- CD3+ CD8- CD4+ CD14- TCRγδ- CD11c-	22.4	21.2	26.2	22.3	26.6
				*Naïve*	CD4 T cells + CD45RA+ CCR7+ CD27+	8.92	9.12	12.9	9.51	15.1
				*Central memory*	CD4 T cells + CD45RA- CCR7+ CD27+	1.60	1.44	2.09	1.96	4.86
				*Effector memory*	CD4 T cells + CD45RA- CCR7- CD27+	10.7	9.79	4.09	2.83	5.12
				*Terminal effector*	CD4 T cells + CD45RA- CCR7- CD27-	1.12	0.86	3.83	2.84	5.10
				**Treg cells**	CD4 T cells + CD25+ CD127- CCR4+	0.71	0.82	0.47	0.39	0.47
				**Th1-like**	CD4 T cells + CXCR3+ CCR6- CXCR5- CCR4-	0.70	0.54	1.11	0.58	1.27
				**Th2-like**	CD4 T cells + CXCR3- CCR6- CXCR5- CCR4+	1.12	2.06	1.33	1.09	1.95
				**Th17-like**	CD4 T cells + CXCR3- CCR6+ CXCR5- CCR4+	2.05	1.94	1.76	1.49	2.43
			**γδ T cells**		CD66b- CD3+ CD8dim,- CD4- CD14- TCRγδ dim,+	0.79	0.87	2.00	1.55	4.04
			CD4^−^ T Cells							
				**MAIT/NKT cells**	CD66b- CD3+ CD4- CD14- CD161+ TCRγδ- CD28+ CD16-	0.12	0.47	0.87	0.635	0.90
		B cells			CD3- CD14- CD56- CD16 dim,- CD19+ CD20+ HLA-DR dim,+	29.4	25.7	11.8	9.67	11.9
			*Naïve*		B cells + CD27-	27.4	23.8	9.19	7.42	10.0
			*Memory*		B cells + CD27+	1.91	1.73	1.82	1.34	2.2
			*Plasmablasts*		CD3- CD14- CD16-,dim CD66b- CD20- CD19+ CD56- CD38++ CD27+	0.11	0.13	0.11	0.09	0.12
		NK cells			CD14- CD3- CD123- CD66b- CD45RA+ CD56 dim,+	4.54	9.23	10.7	6.96	11.1
			*Early*		NK cells + CD57-	2.37	4.58	2.36	1.85	2.62
			*Late*		NK cells + CD57+	2.17	4.66	8.31	5.11	8.4
	Monocytes				CD3- CD19- CD56- CD66b- HLA-DR+ CD11c+	10.8	5.5	7.01	6.40	7.41
		**Classical**			Monocytes + CD14+ CD38+	10.0	4.61	5.75	5.02	6.47
		**Transitional**			Monocytes + CD14 dim CD38 dim	0.72	0.68	0.70	0.60	0.76
		**Nonclassical**			Monocytes + CD14- CD38-	0.05	0.19	0.56	0.35	0.62
	Dendritic cells				pDCs+ mDCs	0.32	0.03	0.33	0.26	0.48
		**Plasmacytoid DCs**			CD3- CD19- CD14- CD20- CD66b- HLA-DR dim,+ CD11c- CD123+	0.00	0.00	0.06	0.03	0.09
		**Myeloid DCs**			CD3- CD19- CD14- CD20- HLA-DR dim,+ CD11c dim,+ CD123- CD16 dim,- CD38 dim,+ CD294- HLA-D	0.32	0.03	0.27	0.23	0.39
									
	Granulocytes				Neutrophils + basophils + eosinophils + CD66b- neutrophils	16.2	20.7	18.3	12.0	27.3
		**Neutrophils**			CD66b dim,+ CD16+ HLA-DR-	14.2	19.7	16.6	10.7	25.4
		**Basophils**			HLA-DR- CD66b- CD123 dim,+ CD38+ CD294+	1.36	0.66	0.62	0.44	0.77
		**Eosinophils**			CD14- CD3- CD19- HLA-DR- CD294+ CD66b dim,+	0.09	0.17	0.87	0.48	2.94
		**CD66b^-^ neutrophils**			CD3- CD19- CD66b- CD56- HLA-DR- CD123- CD45-	0.51	0.13	0.49	0.39	0.73

Cell phenotypes are defined according to the criteria established by Bagwell et al. [13]. Boldface font highlights the classification of leukocytes based on differentiation and function, while italics denote the classification based on maturity stage. CD: cluster of differentiation; DCs: dendritic cells; dim: dimly positive; dim, +: dimly positive to positive; HLA: human leukocyte antigen; MAIT: mucosal-associated invariant T; mDCs: myeloid DCs; NK: natural killer; NKT: natural killer T; pDCs: plasmacytoid DCs; Th: T helper; Tregs: regulatory T cells.

## Data Availability

The data sets used and analyzed during the current study are available from the corresponding author upon reasonable request.

## Supplementary Materials

The following supporting information can be downloaded at: https://www.mdpi.com/article/10.3390/vaccines12080878/s1, Figure S1: Fundus findings and visual field results of the right eye in the acute phase; Figure S2: Fundus findings of perivascular chorioretinal atrophy in the acute phase of the right eye; Figure S3: Fundus findings of perivascular chorioretinal atrophy in the acute phase of the left eye; Figure S4: Fundus findings and visual field results of the right eye in the remission phase; Figure S5: Fundus findings and visual field results of the left eye in the remission phase; Table S1: Hematological findings of a COVID-19 patient with bilateral perivascular chorioretinal atrophy in the acute phase; Table S2: Hematological findings of the patient in the remission phase; Table S3: Serum cytokine levels in each control subject; Table S4: Immune cell populations, phenotypes, and proportions of immune cells among leukocytes in the peripheral blood of each control subject.
