# Supplementary material for: Bilateral Perivascular Chorioretinal Atrophy Resembling Pigmented Paravenous Chorioretinal Atrophy Post COVID-19 Infection: A Case Report and Comprehensive Immune Profiling
Source: Vaccines (Basel). 2024 Aug 2;12(8):878. doi: 10.3390/vaccines12080878 (PMC11360358; doi:10.3390/vaccines12080878)
Supplement: Supplementary file 1 [file vaccines-12-00878-s001.zip › vaccines-3091242-supplementary.pdf]

Supplementary Table S1. Hematological findings of a COVID-19 patient with bilateral perivascular chorioretinal atrophy in the acute phase.

Test item	Result	Unit	Reference range
**Blood count**			
RBC	4.61	x 10^6/μL	3.86 to 4.92
Hb	13.1	g/dL	11.6 to 14.8
Ht	39.6	%	35.1 to 44.4
MCV	85.9	fL	83.6 to 98.2
MCH	28.4	pg	27.5 to 33.2
MCHC	33.1	%	31.7 to 35.3
RDW	13.3	fL	11.6 to 14.0
WBC	4.40	x 10^3 /μL	3.3 to 8.6
Neutrophil	64.2	%	38.5 to 80.5
Lymphocyte	25.2	%	16.5 to 49.5
MONO	6.2	%	2.0 to 10.0
EOSINO	3.7	%	0 to 8.5
BASO	0.7	%	0 to 2.5
Platelet	220	x 10^3 /μL	158 to 348
**Biochemistry**			
T-Bil	0.52	mg/dL	0.2 to 1.2
AST	101	U/L	8 to 30
ALT	138	U/L	5 to 35
LDH	223	U/L	100 to 225
Total protein	7.5	mg/dL	6.5 to 8.2
FBS	135	mg/dL	65 to 110
HbA1c	5.8	%	4.6 to 6.2
BUN	10	mg/dL	8 to 20
Creatinine	0.67	mg/dL	0.44 to 0.78
Na	144	mmol/L	135 to 147
K	3.6	mmol/L	3.5 to 5.0
Cl	105	mmol/L	98 to 108
Ca	9.7	mmol/L	8.5 to 10.3
ACE	11.0	IU/L	7.7 to 29.4
eGFR	71.1	mL/min/1.73m^2	
**Endocrine**			
sIL-2R	314.7	U/mL	157 to 474
**Immunity**			
CRP	≦ 0.3	mg/dL	≦ 0.3
IgG	1723	mg/dL	870 to 1700
IgA	217	mg/dL	110 to 410
IgM	66	mg/dL	35 to 220
RF	< 3	IU/mL	≦ 20
ANA	80	times	< 40
Stain type			
Homogeneous	(-)		
Speckled	(+)		
Nucleolar	(-)		
Centromere	(-)		
Peripheral	(-)		
Granular	(-)		
Nuclear membrane	(-)		
PR3-ANCA	< 1.0	U/mL	< 3.5
MPO-ANCA	< 1.0	U/mL	< 3.5
**Antibody**			
RPR	(-)		(-)
TPHA	(-)		(-)
HBsAg	(-)		(-)
Anti-HCV Ab	0.2		< 1.0
**Coagulation**			
PT			
Time	10	sec	9.9 to 11.8
Activity	108.3	%	80 to 127
PT-INR	0.96		0.9 to 1.1
APTT			
Time	26.3	sec	24.0 to 32.0
Fibrinogen	294	mg/dL	180 to 400
ESR (1 hour)	13	mm	3 to 11

ACE: angiotensin converting enzyme; ALT: alanine aminotransferase; ANA: antinuclear antibody; APTT: activated partial thromboplastin time; ANCA: anti-neutrophil cytoplasmic antibody; AST: aspartate transaminase; BASO: basophils; BUN: blood urea nitrogen; Ca: calcium; Cl: chlorine; COVID-19: coronavirus disease 2019; CRP: C-reactive protein; eGFR: estimated glomerular filtration rate; EOSINO: eosinophil; ESR: erythrocyte sedimentation rate; FBS: fasting blood sugar; Hb: hemoglobin; HbA1c: glycated hemoglobin; HBsAg: hepatitis B surface antigen; HCV: hepatitis C virus; Ht: hematocrit; HSV: herpes simplex virus; Ig: immunoglobulin; K: potassium; LDH: lactate dehydrogenase; Na: sodium; MCH: mean corpuscular hemoglobin; MCHC: mean corpuscular hemoglobin concentration; MCV: mean corpuscular volume; MONO:

monocytes; MPO: myeloperoxidase; PT: prothrombin time; PR3: proteinase 3; PT-INR: prothrombin time-international normalized ratio; RF: rheumatoid factor; RPR: rapid plasma regain; RBC: red blood cell; RDW: red blood cell distribution width; sIL-2R: soluble interleukin-2 receptor; T-BIL: total bilirubin; TPHA: treponema pallidum hemagglutination test; WBC: white blood cells.

Supplementary Table S2. Hematological findings of the patient in the remission phase.

Test item	Result	Unit	Reference range
**Blood count**			
RBC	4.59	x 10^6/μL	3.86 to 4.92
Hb	12.8	g/dL	11.6 to 14.8
Ht	40.1	%	35.1 to 44.4
MCV	87.4	fL	83.6 to 98.2
MCH	27.9	pg	27.5 to 33.2
MCHC	31.9	%	31.7 to 35.3
RDW	13.8	fL	11.6 to 14.0
WBC	3.90	x 10^3 /μL	3.3 to 8.6
Neutrophil	64.7	%	38.5 to 80.5
Lymphocyte	25.8	%	16.5 to 49.5
MONO	6.2	%	2.0 to 10.0
EOSINO	2.8	%	0 to 8.5
BASO	0.5	%	0 to 2.5
Platelet	197	x 10^3 /μL	158 to 348
**Biochemistry**			
T-Bil	0.61	mg/dL	0.2 to 1.2
AST	82	U/L	8 to 30
ALT	130	U/L	5 to 35
LDH	178	U/L	100 to 225
Total protein	6.9	mg/dL	6.5 to 8.2
FBS	82	mg/dL	65 to 110
HbA1c	5.7	%	4.6 to 6.2
BUN	12	mg/dL	8 to 20
Creatinine	0.63	mg/dL	0.44 to 0.78
Na	143	mmol/L	135 to 147
K	4.0	mmol/L	3.5 to 5.0
Cl	107	mmol/L	98 to 108
Ca	9.3	mmol/L	8.5 to 10.3
ACE	10.5	IU/L	7.7 to 29.4
eGFR	76.1	mL/min/1.73m^2	

Test item	Result	Unit	Reference range
**Endocrine**			
sIL-2R	300.4	U/mL	157 to 474
**Immunity**			
CRP	≦ 0.3	mg/dL	≦ 0.3
IgG	1498	mg/dL	870 to 1700
IgA	203	mg/dL	110 to 410
IgM	65	mg/dL	35 to 220
RF	3	IU/mL	≦ 20
ANA	80	times	< 40
Stain type			
Homogeneous	(−)		
Speckled	(+)		
Nucleolar	(−)		
Centromere	(−)		
Peripheral	(−)		
Granular	(−)		
Nuclear membrane	(−)		
PR3-ANCA	< 1.0	U/mL	< 3.5
MPO-ANCA	< 1.0	U/mL	< 3.5
**Antibody**			
RPR	(−)		(−)
TPHA	(−)		(−)
HBsAg	(−)		(−)
Anti-HCV Ab	0.1		< 1.0
**Coagulation**			
PT			
Time	10.3	sec	9.9 to 11.8
Activity	114.8	%	80 to 127
PT-INR	0.94		0.9 to 1.1
APTT			
Time	28.1	sec	24.0 to 32.0
Fibrinogen	278	mg/dL	180 to 400
ESR (1 hour)	11	mm	3 to 11

Abbreviations are defined as shown in the footnote of Supplementary Table S1.

Supplementary Table S3. Serum cytokine levels in each control subject.

Cytokine	Controls			Detection range	
Reference number	No.1	No.2	No.3	Lower	Upper
PDGF-BB	83.6	572.1	874.2	10.5	42619
IL-1β	0.31	0	0	0.34	5037
IL-1ra	0	27.8	24.2	8.56	37277
IL-2	0	0	0	1.63	7740
IL-4	0.27	1.92	3.19	0.17	3540
IL-5	0	0	0	6.02	85349
IL-6	0	0	0	0.98	3825
IL-7	0	0	0	1.84	11702
IL-8	0	1.30	2.17	0.51	10416
IL-9	0	29.9	45.1	2.11	10043
IL-10	0	0	0	2.77	12756
IL-12	0	0	0	1.58	21263
IL-13	0	0	0	0.75	3908
IL-15	0	0	0	559.4	76423
IL-17A	0	0	3.05	2.65	35294
Eotaxin	7.60	5.20	134.7	0.20	787.1
bFGF	4.67	0	0	5.45	5446
G-CSF	0	43.8	111.1	57.3	6793
GM-CSF	0	0	0	0.33	1534
IFN-γ	0	0	0	1.20	22826
IP-10	48.3	70.5	142.1	1.53	23607
MCP-1	0	3.35	6.98	0.34	5762
MIP-1α	0	0.44	0.40	0.05	49.0
MIP-1β	3.41	22.3	20.1	0.46	2126
RANTES	28.6	388.4	150.0	1.00	3499
TNFα	0	4.07	0	4.07	18347
VEGF-A	0	0	0	19.5	61637

Cytokine concentrations are expressed as pg/mL. Levels below the detectable limit were assigned a value of zero [14]. AH: aqueous humor; bFGF: basic fibroblast growth factor; CSF: cerebrospinal fluid; G-CSF: granulocyte colony-stimulating factor; GM-CSF: granulocyte macrophage colony-stimulating factor; IFN-γ: interferon-gamma; IL: interleukin; IL-1ra: IL-1 receptor antagonist; IP-10: interferon gamma-induced protein 10; MCP-1: monocyte chemotactic protein-1; MIP: macrophage inflammatory protein; PDGF: platelet derived growth factor; RANTES: regulated on activation, normal T-cell

expressed and secreted; TNFα: tumor necrosis factor alpha; VEGF: vascular endothelial growth factor.

Supplementary Table S4. Immune cell populations, phenotypes, and proportions of immune cells among leukocytes in the peripheral blood of each control subject.

Populations	Model phenotypes	Controls		
**Reference number**		**No.1**	**No.2**	**No.3**
Intact live cells (%)		100	100	100
Lymphocytes	CD3 T cells + B cells + NK cells + plasmablasts	46.8	76.8	59.8
$CD3^+$ T cells	CD8 T cells + CD4 T cells + γδ T cells + MAIT/NKT cells	28.6	53.5	44.8
$CD8^+$ T cells	CD3+ CD66b- CD19- CD8+ CD4- CD14- CD161- TCRγδ- CD123- CD11c-	8.14	24.9	10.7
Naïve	CD8 T cells + CD45RA+ CCR7+ CD27+	1.62	2.32	4.12
Central memory	CD8 T cells + CD45RA- CCR7+ CD27+	0.10	0.23	0.12
Effector memory	CD8 T cells + CCR7- CD27+	1.83	3.52	2.04
Terminal effector	CD8 T cells + CCR7- CD27-	4.59	18.78	4.46
$CD4^+$ T cells	CD66b- CD3+ CD8- CD4+ CD14- TCRγδ- CD11c-	18.4	26.2	27.1
Naïve	CD4 T cells + CD45RA+ CCR7+ CD27+	12.9	6.10	17.3
Central memory	CD4 T cells + CD45RA- CCR7+ CD27+	2.09	7.62	1.82
Effector memory	CD4 T cells + CD45RA- CCR7- CD27+	1.56	6.15	4.09
Terminal effector	CD4 T cells + CD45RA- CCR7- CD27-	1.84	6.36	3.83
**Treg cells**	CD4 T cells + CD25+ CD127- CCR4+	0.47	0.47	0.31
**Th1-like**	CD4 T cells + CXCR3+ CCR6- CXCR5- CCR4-	0.04	1.11	1.43
**Th2-like**	CD4 T cells + CXCR3- CCR6- CXCR5- CCR4+	1.33	2.56	0.84
**Th17-like**	CD4 T cells + CXCR3- CCR6+ CXCR5- CCR4+	1.22	3.09	1.76
**γδ T cells**	CD66b- CD3+ CD8dim,- CD4- CD14- TCRγδ dim,+	1.10	2.00	6.08
$CD4^-$ T Cells				
**MAIT/NKT cells**	CD66b- CD3+ CD4- CD14- CD161+ TCRγδ- CD28+ CD16-	0.92	0.40	0.87
B cells	CD3- CD14- CD56- CD16 dim,- CD19+ CD20+ HLA-DR dim,+	7.52	11.9	11.8
Naïve	B cells + CD27-	5.64	9.19	10.9
Memory	B cells + CD27+	1.82	2.59	0.85
Plasmablasts	CD3- CD14- CD16-,dim CD66b- CD20- CD19+ CD56- CD38++ CD27+	0.06	0.13	0.11
NK cells	CD14- CD3- CD123- CD66b- CD45RA+ CD56 dim,+	10.7	11.4	3.24
Early	NK cells + CD57-	2.36	2.87	1.33
Late	NK cells + CD57+	8.31	8.55	1.91
Monocytes	CD3- CD19- CD56- CD66b- HLA-DR+ CD11c+	5.78	7.01	7.81
**Classical**	Monocytes + CD14+ CD38+	4.29	5.75	7.18
**Transitional**	Monocytes + CD14 dim CD38 dim	0.81	0.70	0.49
**Nonclassical**	Monocytes + CD14- CD38-	0.68	0.56	0.14
Dendritic cells	pDCs+ mDCs	0.63	0.33	0.18
**Plasmacytoid DCs**	CD3- CD19- CD14- CD20- CD66b- HLA-DR dim,+ CD11c- CD123+	0.12	0.06	0
**Myeloid DCs**	CD3- CD19- CD14- CD20- HLA-DR dim,+ CD11c dim,+ CD123- CD16 dim,- CD38 dim,+ CD294- HLA-D	0.51	0.27	0.18
Granulocytes	Neutrophils + basophils + eosinophils + CD66b- neutrophils	36.3	5.64	18.3
**Neutrophils**	CD66b dim,+ CD16+ HLA-DR-	34.3	4.84	16.6
**Basophils**	HLA-DR- CD66b- CD123 dim,+ CD38+ CD294+	0.91	0.26	0.62
**Eosinophils**	CD14- CD3- CD19- HLA-DR- CD294+ CD66b dim,+	0.87	5.00	0.09
**$CD66b^-$ neutrophils**	CD3- CD19- CD66b- CD56- HLA-DR- CD123- CD45-	0.29	0.49	0.97

Cell phenotypes are defined according to the criteria established by Bagwell et al. [13]. Boldface font highlights the classification of leukocytes based on differentiation and function, while italics denote classification based on maturity stage. CD: cluster of differentiation; DCs: dendritic cells; dim: dimly positive; dim,+: dimly positive to positive; HLA: human leukocyte antigen; MAIT: mucosal-associated invariant T; mDCs: myeloid DCs; NK: natural killer; NKT: natural killer T; pDCs: plasmacytoid

DCs; Th: T helper; Tregs: regulatory T cells.

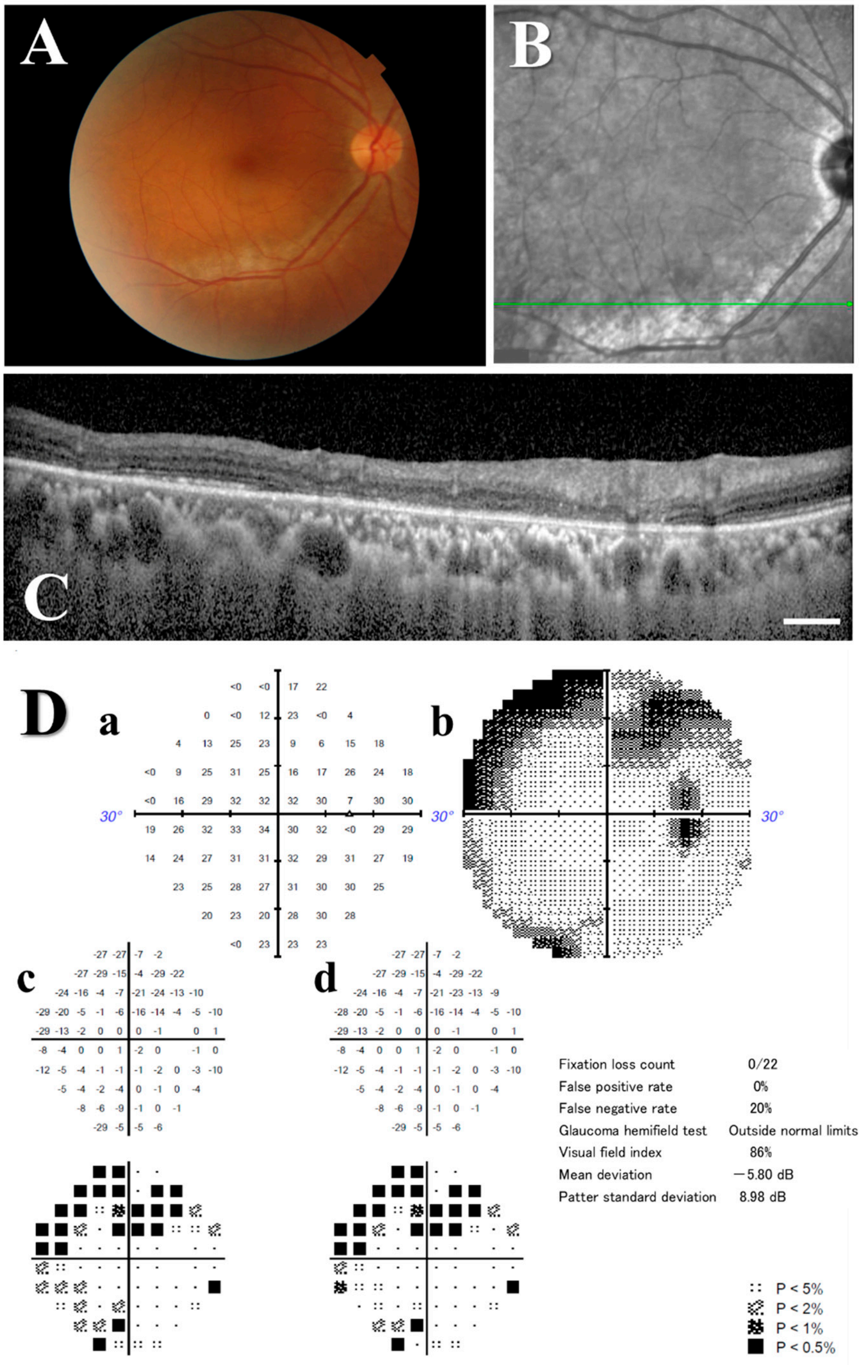

Supplementary Figure S1. Fundus findings and visual field results of the right eye in the acute phase. (**A**) Color fundus photograph shows absence of retinal pigment along the inferior arcade vessels extending from the disc to the mid-peripheral retina,

suggesting perivascular chorioretinal atrophy associated with COVID-19. (**B**) Red-free fundus photograph and (**C**) cross-sectional EDI-OCT image of the retina along the green line. The outer retinal layers are thinned, and the retinal layers are indistinguishable. The ellipsoid zone (photoreceptor layer) in the outer retinal layer is absent. Scale bar (white horizontal bar): 200 µm. (**D**) A Humphrey 30-2 SITA-Standard visual field test demonstrates scotomas in the superior temporal quadrant corresponding to the lesions with mean deviation of −5.80 dB. (**a**) numerical sensitivity plot, (**b**) grayscale map, (**c**) total deviation map, and (**d**) pattern deviation map are shown. COVID-19: coronavirus disease 2019; EDI-OCT: enhanced depth imaging optical coherence tomography; SITA: Swedish interactive thresholding algorithm.

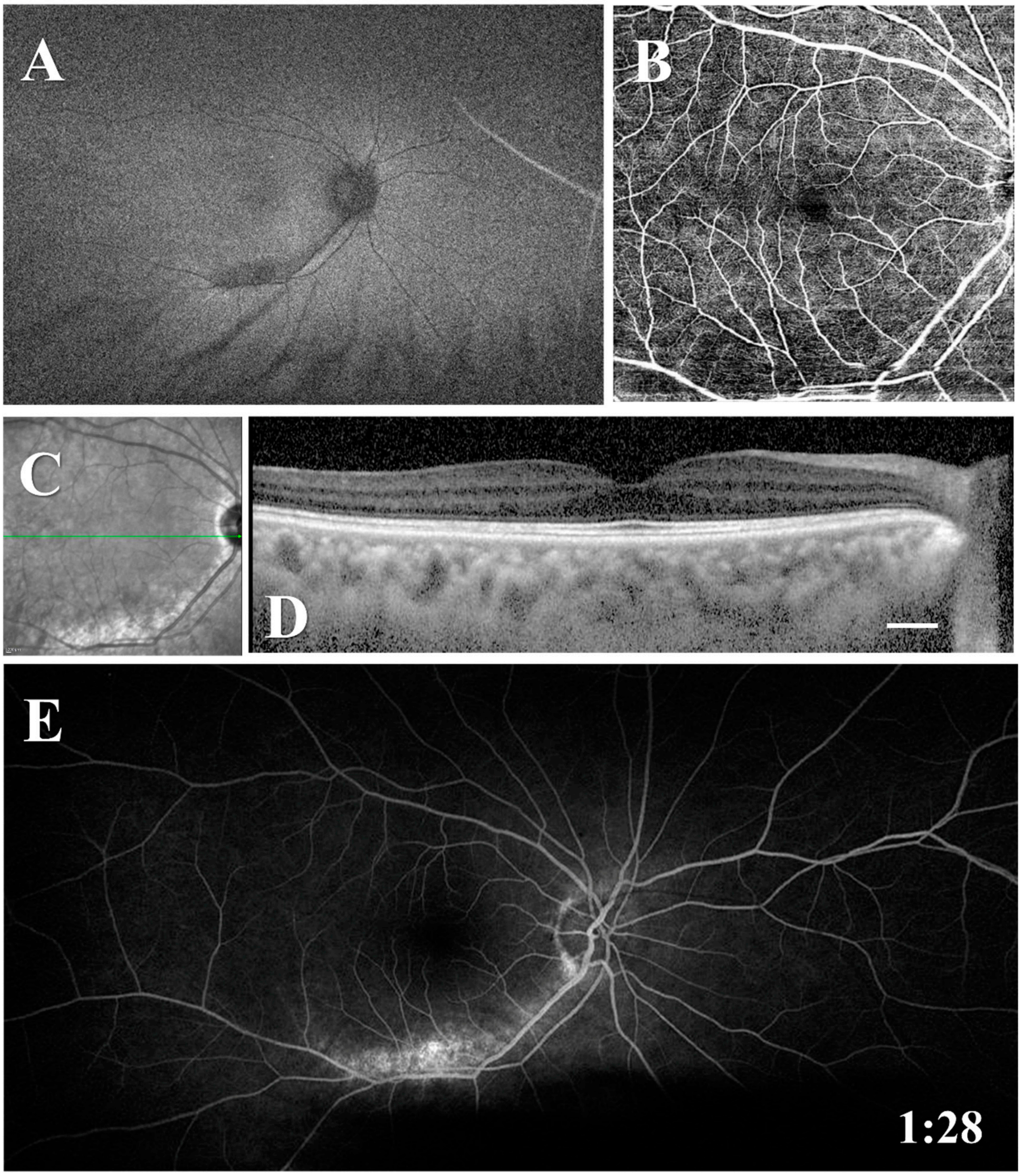

Supplementary Figure S2. Fundus findings of perivascular chorioretinal atrophy in the acute phase of the right eye. (**A**) FAF image demonstrates hypofluorescence of the RPE layer in the lesions, suggesting RPE cell dysfunction. (**B**) OCTA of the entire macula reveals preserved retinal microcirculation within the lesions. (**C**) Red-free fundus photograph and (**D**) cross-sectional EDI-OCT image of the retina at the fovea show no

remarkable abnormalities in anatomical structure. Scale bar (white horizontal bar): 200 µm. (**E**) FA image exhibits multiple punctate hyperfluorescent spots along the lesions (white dots), indicating focal RPE defects, but no leakage and pooling. Time of photography after administration of FA is shown in the lower right corner. EDI-OCT: enhanced depth imaging optical coherence tomography; FA: fluorescein angiography; FAF: fundus autofluorescence; OCTA: optical coherence tomography angiography; RPE: retinal pigment epithelium.

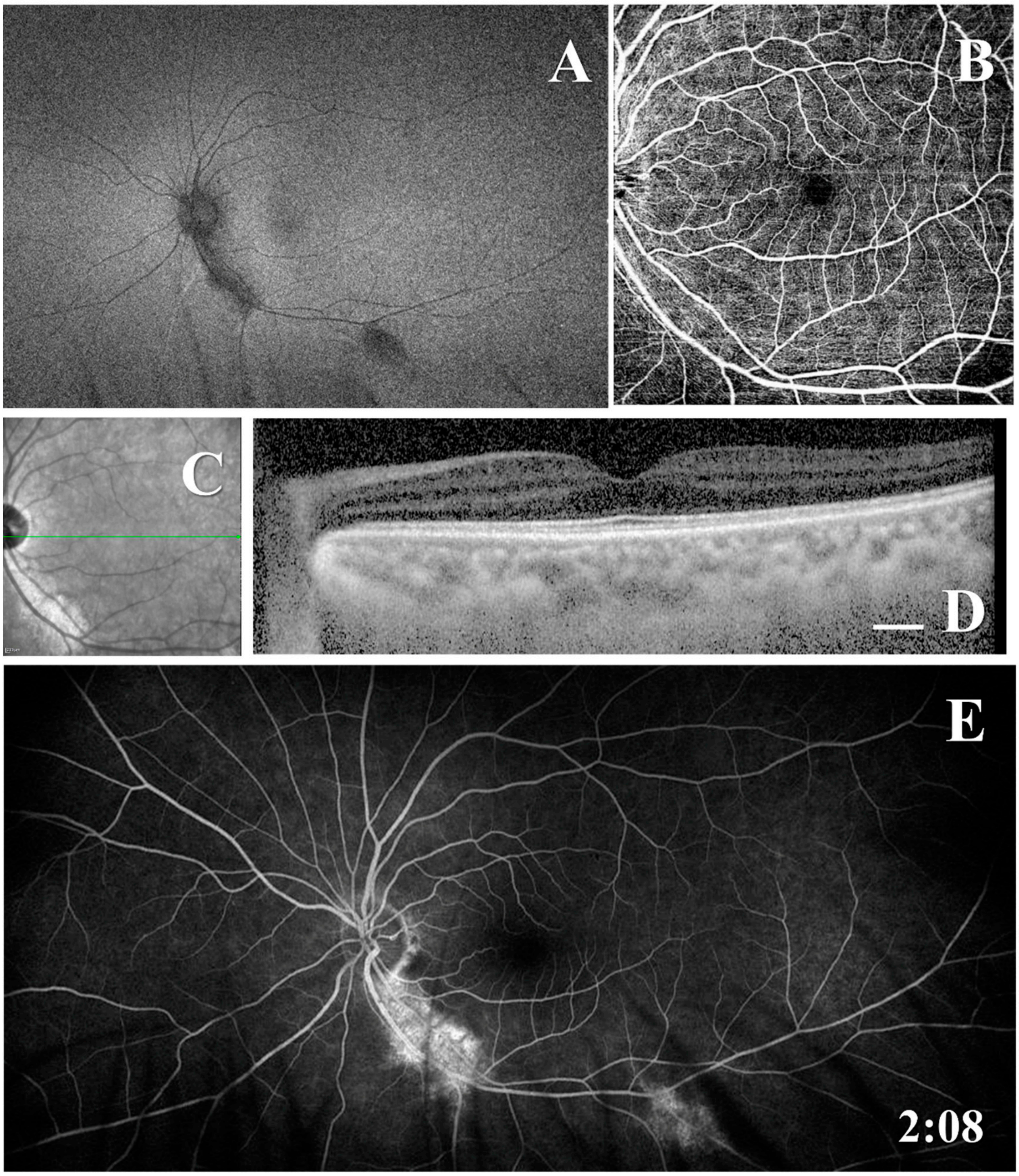

Supplementary Figure S3. Fundus findings of perivascular chorioretinal atrophy in the acute phase of the left eye. (**A**) FAF image, (**B**) OCTA image of the entire macula, (**C**) red-free fundus photograph, (**D**) tomographic photograph corresponding to fovea by EDI-OCT scan, and (**E**) FA image are shown. Abbreviations are defined as shown in the footnote of Supplementary Figure S2.

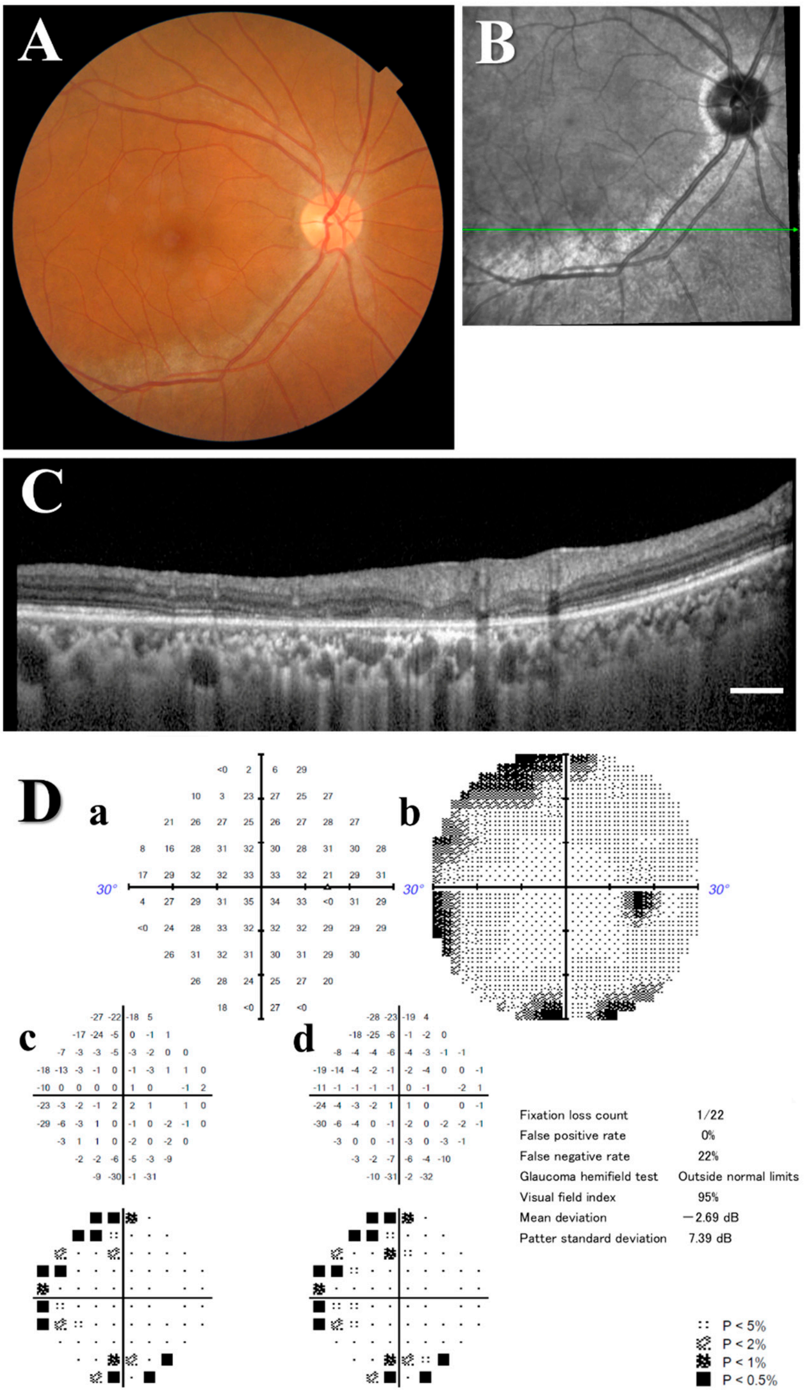

Supplementary Figure S4. Fundus findings and visual field results of the right eye in the remission phase. (**A**) Color fundus photograph shows waxy white lesions along retinal blood vessels. Compared to the acute phase, there is no significant expansion in the area of the lesions. (**B**) Red-free fundus photograph and (**C**) cross-sectional EDI-

OCT image of the retina along the green line. Thinning of the outer retina is reduced, and the anatomical structure of the outer retinal layers recovers and becomes distinguishable. Scale bar (white horizontal bar): 200 μm. (**D**) A Humphrey 30-2 SITA-Standard visual field test demonstrates improvement with mean deviation of −2.69 dB in visual field defects (scotomas) observed in the acute phase. (**a**) numerical sensitivity plot, (**b**) grayscale map, (**c**) total deviation map, and (**d**) pattern deviation map are shown. Abbreviations are defined as shown in the footnote of Supplementary Figure S1.

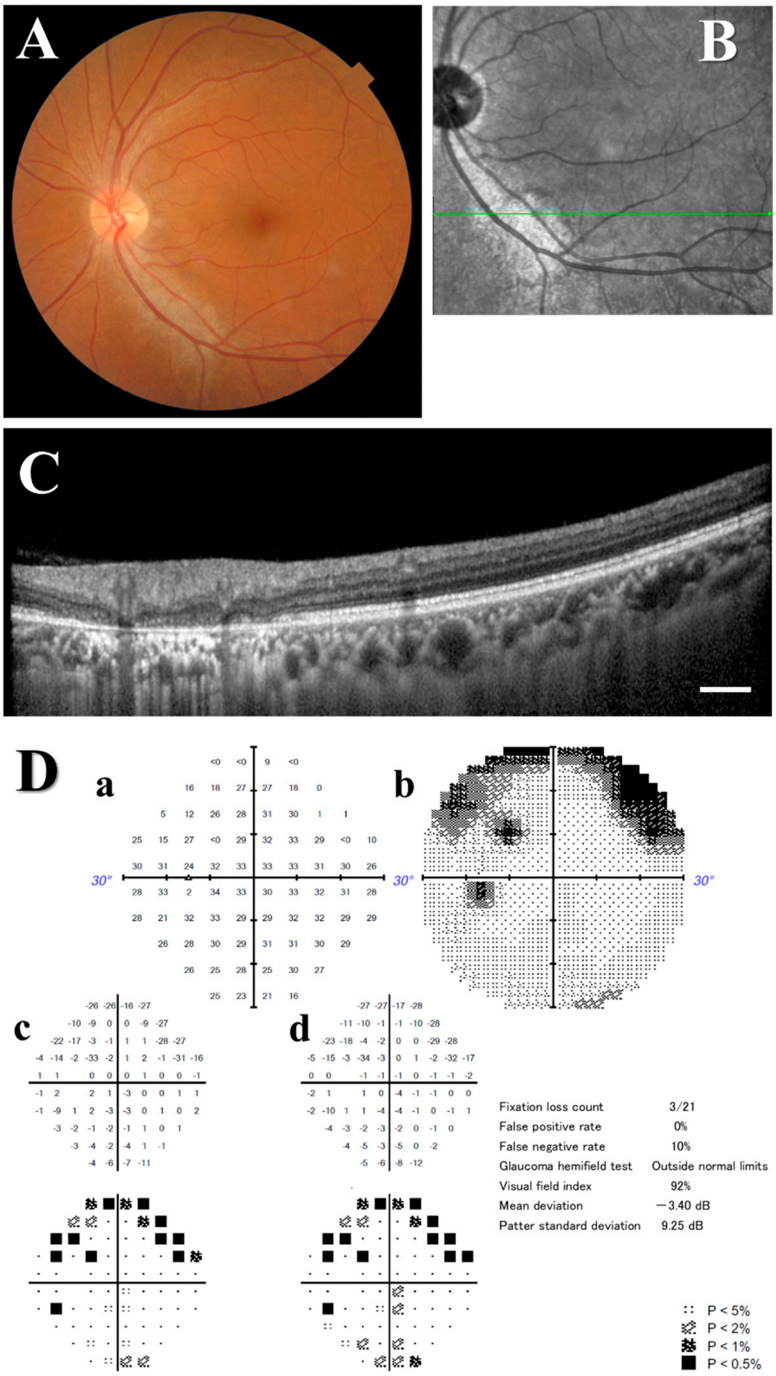

Supplementary Figure S5. Fundus findings and visual field results of the left eye in the remission phase. (**A**) Color fundus photograph, (**B**) red-free fundus photography, (**C**) cross-sectional EDI-OCT image of the retina along the green line. (**D**) A Humphrey 30-

2 SITA-Standard visual field test demonstrates improvement with mean deviation of −3.40 dB in visual field defects (scotomas) observed in the acute phase. (**a**) numerical sensitivity plot, (**b**) grayscale map, (**c**) total deviation map, and (**d**) pattern deviation map are shown. Abbreviations are defined as shown in the footnote of Supplementary Figure S1.
